# The impact of public coverage of newer hepatitis C medications on utilization, adherence, and costs in British Columbia

**DOI:** 10.1371/journal.pone.0247843

**Published:** 2021-03-01

**Authors:** Harriet Ho, Naveed Z. Janjua, Kimberlyn M. McGrail, Mark Harrison, The BC Hepatitis Testers Cohort Team, Michael R. Law

**Affiliations:** 1 The Centre for Health Services and Policy Research, School of Population and Public Health, The University of British Columbia, Vancouver, British Columbia, Canada; 2 British Columbia Centre for Disease Control, Vancouver, British Columbia, Canada; 3 School of Population and Public Health, The University of British Columbia, Vancouver, British Columbia, Canada; 4 Centre for Health Evaluation and Outcomes Sciences, St. Paul’s Hospital, Vancouver, British Columbia, Canada; 5 Faculty of Pharmaceutical Sciences, The University of British Columbia, Vancouver, British Columbia, Canada; Kaohsiung Medical University, TAIWAN

## Abstract

**Background:**

Sofosbuvir and ledipasvir-sofosbuvir are both newer direct-acting antiviral agents for the treatment of hepatitis C. The high list prices for both drugs have led to concern about the budget impact for public drug coverage programs. Therefore, we studied the impact of public prescription drug coverage for both drugs on utilization, adherence, and public and private expenditure in British Columbia, Canada.

**Methods:**

We used provincial administrative claims data from January 2014 to June 2017 for all individuals historically tested for either hepatitis C and/or human immunodeficiency virus. Using interrupted time series analysis, we examined the impact of public insurance coverage on treatment uptake, adherence (proportion of days covered), and public and private expenditures.

**Results:**

Over our study period, 4,462 treatment initiations were eligible for analysis (1,131 sofosbuvir and 3,331 ledipasvir-sofosbuvir, which include 19 patients initiated on both treatments). We found the start of public coverage for sofosbuvir and ledipasvir-sofosbuvir increased treatment uptake by 154%. Adherence rates were consistently high and did not change with public coverage. Finally, public expenditure increased after the policy change, and crowded out some private expenditure.

**Conclusion:**

Public coverage for high-cost drugs for hepatitis C dramatically increased use of these drugs, but did not reduce adherence. From a health policy perspective, public payers should be prepared for increased treatment uptake following the availability of public coverage. However, they should not be concerned that populations without private insurance coverage will be less adherent and not finish their treatment course.

## Introduction

Specialty pharmaceuticals are a designation of drugs that are considered high-cost [1]. Spending on specialty pharmaceuticals is expected to grow in the coming years and is projected to constitute 50 percent of health plans’ overall drug spending by 2019, making them a major issue for both public and private payers [2]. Sofosbuvir (SOF) and ledipasvir/sofosbuvir (LDV/SOF) are two newer specialty drugs that treat hepatitis C virus (HCV) with a high cure rate. The high costs of these therapies have been very controversial, with a once-a day, eight- to 24-week course of treatment costing, on average, $60,000 per patient [3]. This has led to concern from both public and private insurers about the cost implications given the prevalence of HCV in the population. There has been limited study on the impact of public coverage of direct acting antivirals (DAAs), such as SOF and LDV/SOF, on treatment uptake and expenditure. One study conducted in the US examined found that Medicaid coverage of SOF led to a short-lived increase in prescription volume and expenditures within the program [4]. However, it is unclear what the impact of coverage of SOF had at the population level.

A related consideration is whether broader public coverage, including for at-risk patients, will have implications for adherence to prescriptions. Adherence is crucial to the effectiveness of DAAs to obtain maximum response to treatment, avoid treatment failure, and prevent resistance [5]. With the high cost of SOF and LDV/SOF, ensuring that treatment achieves the best outcomes through high adherence is one way to maximize value for money. To date there has been very little study of patient adherence to new DAAs outside of clinical trials [6–9]. In other drugs, decreased out-of-pocket costs were associated with higher adherence [10, 11]. However, SOF and LDV/SOF have short, defined treatment courses, which may see different effects of coverage on adherence, compared to drugs taken for more extended durations, such as statins, in these prior studies. Further, it is unclear whether adherence behaviours will be different between populations with private coverage and those who received public coverage under the expansion.

In the absence of good data on the outcomes and cost implications of coverage, different insurers have been imposing different clinical criteria on coverage for these medications. For example, British Columbia’s (BC) public PharmaCare program introduced coverage for SOF and LDV/SOF more than a year after the first prescription of SOF was dispensed in BC [12]. This coverage was initially made with strict clinical criteria including fibrosis at stage F2 or higher [3]. As a growing number of new and expensive specialty medicines becomes available, the impact of public listing becomes an important consideration for both public drug plans and employers providing private drug coverage in Canada. To provide insight about the impact of this coverage, we used rigorous longitudinal methods to analyze trends in adherence, treatment uptake, and expenditure for SOF and LDV/SOF following public coverage.

## Materials and methods

### Study context

This analysis was part of a larger project that was granted ethics approval by the University of British Columbia Research Ethics Board (#: H15-01776). Participant consent was not required because analysis was performed on the de-identified data.

BC’s health care system consists of a single-payer system for physician and hospital services, with a mix of public and private payers for prescription drugs dispensed outside of hospitals [13]. Under PharmaCare, BC residents can enrol in one of several plans, the largest of which is the income-based Fair PharmaCare plan [14]. This plan requires most households to spend a certain proportion of their household income as a deductible before public coverage commences. There are also other plans with more extensive subsidy, such as a plan for recipients of social assistance payments. PharmaCare coverage of SOF and LDV/SOF commenced in March 2015 [12]. Prior to this, these drugs could be accessed by using private insurance plans (where they chose to provide coverage) or out-of-pocket payments by individuals. Of note, even after public listing, private insurance plans could still cover the portion of the drug cost under any applicable public plan deductible or for individuals who do not meet the public plan’s clinical criteria.

### Data sources

Our analysis studied the BC Hepatitis Testers Cohort, which is housed at the BC Centre for Disease Control. This cohort includes all individuals in BC who have been tested for HCV or HIV, or reported a case of hepatitis B (HBV), HCV, HIV or active tuberculosis in BC from 1990 to February 2016 [15]. Through personal health numbers, the data are linked to medical visits, hospitalizations, prescription drugs, the cancer registry and mortality data [15].

Healthcare utilization data came from comprehensive sources that capture nearly all BC residents’ health system usage, including diagnoses, medical and surgical procedures, and prescription drug dispensations. Prescription drug data came from PharmaNet, which is a network run by the BC Ministry of Health and the College of Pharmacists of BC [16]. PharmaNet provides comprehensive data on all prescription drugs dispensed through community pharmacies, community health practices, and hospital outpatient pharmacies in BC, regardless of payer [17].

### Inclusion criteria

Our analysis examined usage of SOF and LDV/SOF between January 2014 and June 2017. For our analyses of adherence and uptake, we included all initiations prior to January 14, 2017 that had a treatment length not exceeding 168 days to allow adequate time for follow-up of the complete course of treatment.

### Statistical analysis

We used interrupted time series (ITS) analysis, which is one of the most robust quasi-experimental study designs that has been used extensively in evaluating drug policy changes [18]. We examined monthly time points; for the adherence and treatment uptake analyses, the monthly time points were the starting month of treatment for a patient. As PharmaCare coverage began on March 23, 2015, we classified March 2015 as being in the pre-period as the majority of the month fell into that time period. Since the effects of the coverage changes may not be immediate, we used a three-month phase-in period in our analyses by excluding the data points for three months following the announcement (April to June 2015).

We examined three key outcome variables in our ITS analyses:

- **Number of individuals treated–**The total number of individuals who started treatment in each month.
- **Adherence–**Proportion of days covered (PDC) measures the percentage of days that medication is available and is capped at 100%. The total number of days evaluated can either be the treatment duration or a defined study period (e.g., 30 days, 90 days) for a chronic disease treatment. As the recommended durations of treatments are 8, 12, 16, and 24 weeks, we defined treatment periods in the equivalent number of days: 56, 84, 112, or 168 days. We identified treatment courses based on quantity of pills dispensed, rounded up to the next treatment course. Treatment duration is dependent on factors, including genotype of HCV [19]. We examined the average PDC at each month, weighted by the number of individuals in that month.

PDC=(totaldayssupply/totalnumberofdaysevaluated)×100%

- **Public and private expenditure–**The total amounts of public and private expenditure in each month.

In all of our analyses, we used generalized least squares (GLS) models with appropriate adjustment for autocorrelation [20]. All analyses were conducted using SAS Version 9.4 and R Version 3.3.2.

## Results

### Cohort characteristics

Our cohort contained 4,462 initiations of SOF and LDV/SOF during our study period (Table 1). Among these treatments, 19 individuals were treated twice (once with each treatment); thus, the cohort contained 4,443 unique individuals. The average age was 57.9 years, and 32.3% were female. Of those treated with SOF or LDV/SOF, 93.3% were white, 43.9% belonged to the least privileged category of material deprivation, 4.7% had an injection drug use diagnosis, and 15.5% had cirrhosis.

**Table 1:** Characteristics of eligible patients treated on sofosbuvir and ledipasvir-sofosbuvir in British Columbia^a^.

Variable	n = 4,443	%
Treatment type		
Sofosbuvir^b^	1,112	25.0
Ledipasvir-sofosbuvir^b^	3,312	74.5
Sofosbuvir and ledipasvir-sofosbuvir	19	0.4
Sex		
Female	1,434	32.3
Male	3,009	67.7
Age		
< 50	695	15.6
50–60	1,713	38.6
> 60	2,035	45.8
Ethnicity		
Non-White	268	6.0
White	4,146	93.3
Unknown	29	0.7
PharmaCare		
No	1,081	24.3
Yes	3,362	75.7
Treatment duration		
56 days	908	20.4
84 days	2,277	51.2
112 days	73	1.6
168 days	1,185	26.7
Previous treatment		
No	3,258	73.3
Yes	1,185	26.7
Material deprivation		
Most privileged	1,501	33.8
Moderately privileged	902	20.3
Least privileged	1,951	43.9
Unknown/missing	89	2.0
Elixhauser comorbidity index		
0	1,479	33.3
1	986	22.2
≥ 2	1,978	44.5
Cirrhosis		
No	3,755	84.5
Yes	688	15.5
Diabetes		
No	3,947	88.8
Yes	496	11.2
Hepatitis B (HBV)		
No	4,128	92.9
Yes	315	7.1
Hepatocellular carcinoma (HCC)		
No	4,387	98.7
Yes	56	1.3
Major mental illness		
No	3,065	69.0
Yes	1,378	31.0
Injection drug use (IDU) diagnosis		
No	4,232	95.3
Yes	211	4.7
Opioid substitution therapy (OST)		
No	4,153	93.5
Yes	290	6.5
Problem alcohol use		
No	3,280	73.8
Yes	1,163	26.2
HIV		
No	4,042	91.0
Yes	401	9.0
Chronic kidney disease (CKD)		
No	4,430	99.7
Yes	13	0.3
End-stage renal disease (ESRD)		
No	4,435	99.8
Yes	8	0.2

^a^ Patients were initiated on sofosbuvir or ledipasvir-sofosbuvir between January 2014 and January 2017.

^b^ Patient counts for sofosbuvir and ledipasvir-sofosbuvir respectively exclude those who were initiated on both treatments.

While on SOF, patients took an average of 4.46 other drugs, with 14.9% taking interferon, 82.8% taking ribavirin, and 14.9% taking both interferon and ribavirin (Table 2). While on LDV/SOF, patients took fewer other drugs. Patients took an average of 3.05 other drugs, with 0.1% taking interferon, 2% taking ribavirin and, and 0.1% taking both interferon and ribavirin.

**Table 2:** Drugs taken while on treatment for sofosbuvir and ledipasvir-sofosbuvir^a^.

Variable	Sofosbuvir	Ledipasvir-sofosbuvir
	n = 1131	n = 3331
Average number of drugs per day,^b^ mean	4.46	3.05
Interferon and ribavirin use, n (%)		
Interferon	169 (14.9)	4 (0.1)
Ribavirin	937 (82.8)	66 (2)
Interferon and ribavirin^c^	169 (14.9)	3 (0.1)

^a^ Patients were initiated on sofosbuvir or ledipasvir-sofosbuvir between January 2014 and January 2017.

^b^ Any other drug, excluding sofosbuvir or ledipasvir-sofosbuvir.

^c^ Counts for patients who took interferon and ribavirin were also counted in the separate counts for interferon and ribavirin.

### Treatment uptake

**Fig 1:**
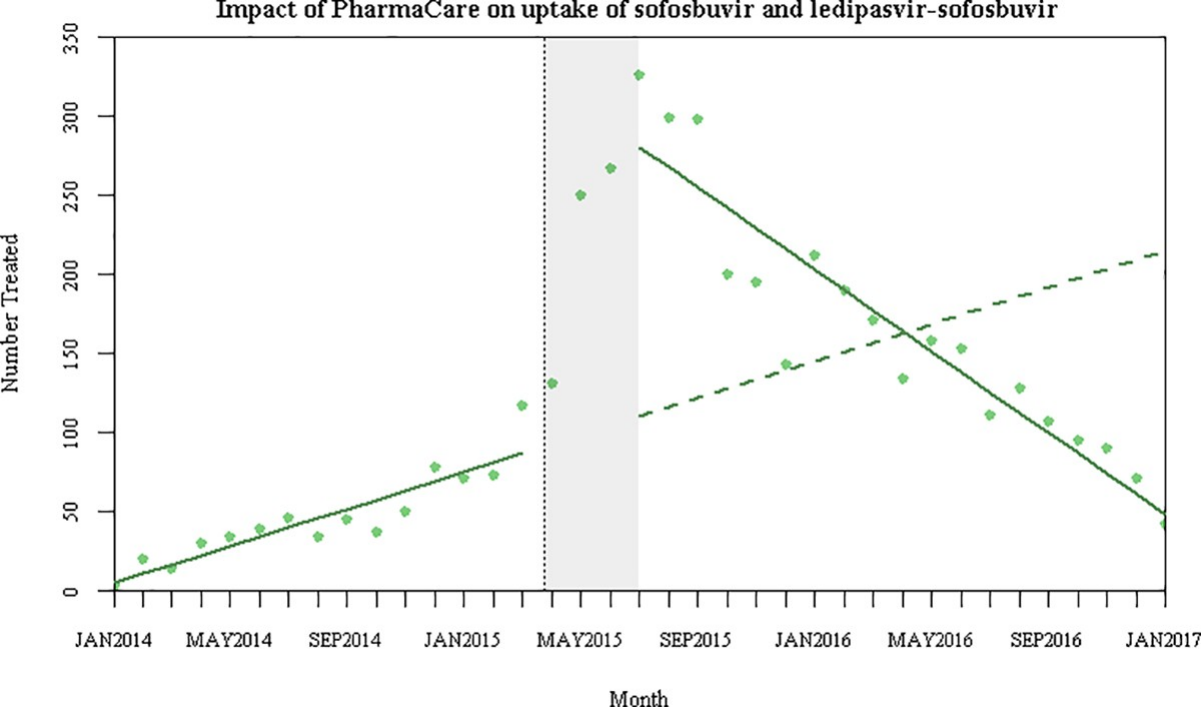
Interrupted time series model for the impact of PharmaCare on treatment uptake of sofosbuvir and ledipasvir-sofosbuvir. The last data point (January 2017) only covered a partial month as the last treatment initiation was January 14, 2017. Thus, there may be small adjustments to the model had we included data for that entire month.

After the institution of public coverage, there was a sustained increase of 189 new individuals treated per month (p < 0.001), followed by a decrease in treatment uptake of 19 individuals per month (p < 0.001) (Table 3 and Fig 1). Over the entire follow-up period, this represented a net increase of just 27 people compared to the counterfactual.

**Table 3:** Results from interrupted time series analyses for the impact of PharmaCare on treatment uptake, adherence and monthly expenditure of sofosbuvir and ledipasvir-sofosbuvir.

Treatment uptake^a^	Level (n persons) (95% CI^b^)	p-value	Slope (n persons) (95% CI)	p-value
	189 (151, 227)	< 0.001***	-19 (-22, -15)	< 0.001***
**Adherence**^**a**^	**Level (PDC**^**c**^ **%) (95% CI)**	**p-value**	**Slope (PDC %) (95% CI)**	**p-value**
	-0.0016 (-0.026, 0.023)	0.90	-0.0015 (-0.004, 0.001)	0.29
**Monthly expenditure**^**d**^	**Level ($) (95% CI)**	**p-value**	**Slope ($) (95% CI)**	**p-value**
Public	21,355,361, (19,525,941, 23,184,782)	< 0.001***	-842,053 (-999,267, -684,839)	< 0.001***
Private	-3,835,460 (-4,531,728, -3,139,193)	< 0.001***	-417,528 (-470,932, -364,124)	< 0.001***

^a^ Includes data points from January 2014 to January 2017.

^b^ CI: confidence interval.

^c^ PDC: proportion of days covered.

^d^ Includes data points from January 2014 to June 2017.

### Adherence

**Fig 2:**
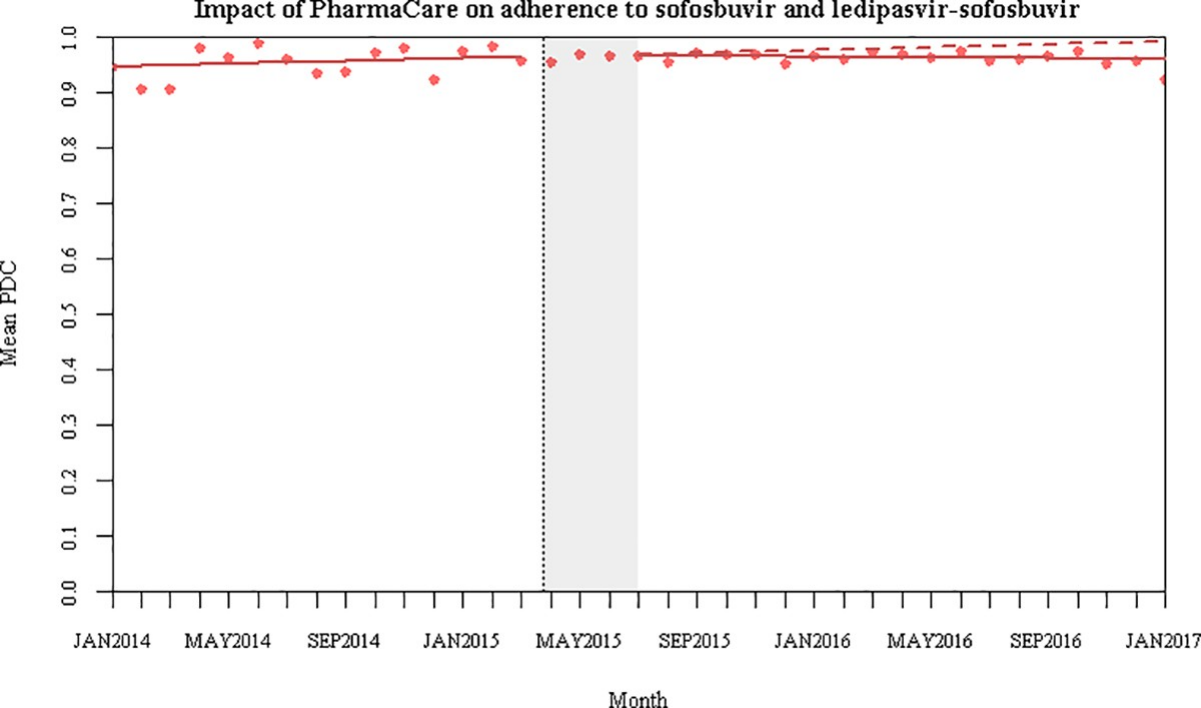
Interrupted time series model for the impact of PharmaCare on adherence to sofosbuvir and ledipasvir-sofosbuvir. The last data point (January 2017) only covered a partial month as the last treatment initiation was January 14, 2017. Thus, there may be small adjustments to the model had we included data for that entire month. PDC: proportion of days covered.

Adherence rates in the period before public coverage were high, with an average PDC of 95.8% across these 15 months. These levels remained high following the coverage change (Fig 2). Neither the change in level nor trend were significantly different from zero for these drugs, suggesting that PharmaCare coverage did not reduce these high adherence rates.

### Public and private expenditure

**Fig 3:**
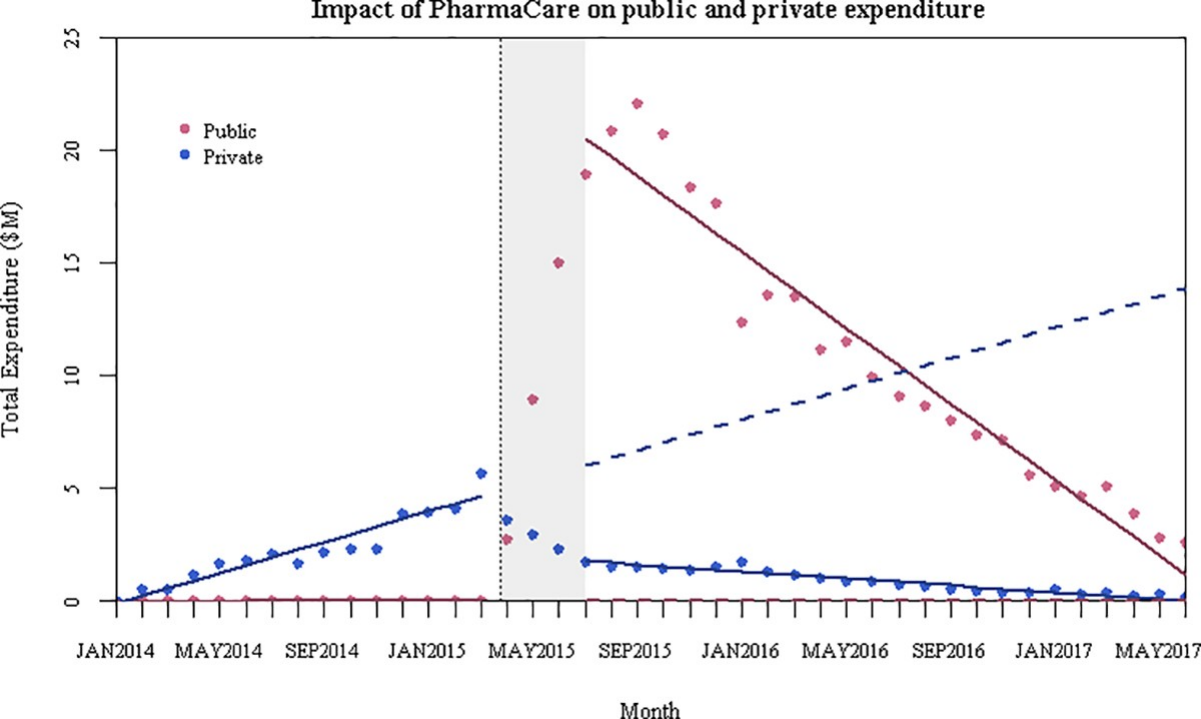
Interrupted time series model for the impact of PharmaCare on public and private expenditure on sofosbuvir and ledipasvir-sofosbuvir.

Looking across all use of the study drugs (including both initiations and other users), we found an immediate increase of $21.36 million in average monthly public expenditure (p < 0.001, 95% CI: $19.53 million, $23.18 million), and a decrease of $842,053 per month thereafter (p < 0.001, 95% CI: -$999,267, -$684,839) (Fig 3). After the policy change, there was a sustained decrease of $3.84 million in average monthly private expenditure (p < 0.001, 95% CI: -$4.53 million, -$3.14 million), followed by a $417,528 decrease per month (p < 0.001, 95% CI: -$470,932, -$364,124). Overall, this suggests that public coverage crowded out at least a portion of the private payments that would have been expected absent the public listing.

## Discussion

### Overview of findings

Over the past few years, the price of specialty drugs and their impact on public sector drug budgets has increased substantially [2]. We found that while the introduction of PharmaCare coverage for SOF and LDV/SOF increased treatment uptake substantially in the short-term, it did not appear to impact adherence rates to these drugs. At the same time, PharmaCare coverage rose and crowded out some of the private expenditure for SOF and LDV/SOF. Taken together, this suggests that public drug plans should prepare for significant increases in use after covering similar medications, but should not be concerned about lower adherence rates in the populations they serve.

Since prior studies have shown that decreased out-of-pocket costs were associated with higher adherence in other drugs [10, 11], one might expect that the availability of PharmaCare coverage would increase adherence rates. However, the lack of an effect of PharmaCare coverage on adherence found in this study may be due to the result of high pre-existing adherence levels. Two factors were likely at play in this result. First, adherence rates for SOF and LDV/SOF were already high prior to the PharmaCare policy change. This may reflect the short duration of treatment and the severity of leaving HCV untreated. However, the overall high adherence rates across the pre- and post-periods were favourable, demonstrating that patients appeared to be taking the drugs for the full duration. Second, the prices of these drugs were beyond the financial means of most residents to pay out-of-pocket prior to public coverage.

There was a rapid surge in treatment uptake following public coverage followed by a decline in treatment uptake. This surge was likely because patients were waiting for the government to eventually cover the drugs. The overall downward trend of uptake after the PharmaCare policy change was expected, as there would have eventually been fewer people left to be treated that would meet the eligibility criteria. These trends aligned with Liao and Fischer’s findings for Medicaid, where they also demonstrated a large increase in uptake in the beginning, which eventually slowed or stabilized [4]. Similar initial surge in treatment uptake following public coverage and decline thereafter has also been observed in Australia [21, 22]. When PharmaCare expands its program to cover individuals with lower severity of HCV [23], we would expect to see similar trends: a spike in treatment uptake when eligibility criteria become less strict, and an overall decrease in uptake over time as there are fewer diagnosed individuals who are already linked with care left in the treatment pool. Sustained treatment uptake will require increasing diagnosis rates.

The increase in public expenditure also aligned with Liao and Fischer’s findings for Medicaid [4]. Large public expenditure was also previously seen in Australia, for a government-funded program for DAAs. Although the Australian government had budgeted $1 billion AUD over 5 years for DAAs, the expenditures in the first 18 months of the program approximated to $3.6 billion AUD; however, the exact direct expenditures are unknown due to a lack of transparency of rebates from manufacturers [24].

### Limitations

Although ITS analysis is one of the most robust quasi-experimental designs, there are some limitations to our study worth noting. First, the PharmaNet data did not allow us to stratify private payments into insurance and out-of-pocket amounts. Therefore, the numbers we reported in this study included individual out-of-pocket payments (either paying for the treatment entirely on their own or co-payment) and the amount covered by private insurance. Second, we do not have a source of data on the criteria for coverage in the many private plans in BC. Thus, it was not possible to determine if differences in criteria drove any of the results.

## Conclusions

Overall, PharmaCare coverage increased public expenditure and resulted in more individuals being able to access treatment. Reassuringly, the effect of public coverage on adherence was not significant when examined over time, and high adherence rates were maintained throughout our study period. This is a promising result, as it suggests public coverage does not result in an increase in poorer adherence to these expensive medicines. With the expansion of coverage in BC and coverage expansions in other jurisdictions, public payers should expect a significant increase in treatment uptake and public costs over the short term and plan resources accordingly.
